# Obituary – Jan Bureš

**DOI:** 10.3389/fnbeh.2012.00062

**Published:** 2012-09-17

**Authors:** André A. Fenton, Lynn Nadel

**Affiliations:** ^1^New York UniversityNew York, NY, USA; ^2^University of ArizonaArizona, USA

A commentary on

Jan Bureš, an exceptionally humane, creative, and gifted experimentalist who died on August 24, 2012 at the age of 86, is rightly viewed as one of the founding fathers of modern neuroscience. Jan was born June 13, 1926 in the Czech Republic, and soon after studying medicine, established with Olga Burešova, his wife and lifelong collaborator, the Laboratory of Neurophysiology of Memory in the Institute of Physiology of the Academy of Sciences in Prague. The laboratory was to become internationally renowned as an intellectual oasis and a hub of innovation.

Already in the 1950s, at a quite young age, Jan Bureš had made his mark, first with his doctoral work on epilepsy and then with his seminal research on cortical spreading depression. Jan and Olga brought that phenomenon under experimental control, worked out the mechanism, then by using it to temporarily inactivate brain regions during select phases of learning, they pioneered the concept of a reversible brain lesion that remains central to contemporary attempts to dissect the brain circuits of learning and memory.

Jan participated in the famous Moscow Colloquium (1958), and his chapter in the 1960 volume helped bring his work to the attention of a western audience. Not long after that, young scientists from the west came to Prague to do postdoctoral work with Jan, establishing an international flavor that permeated laboratory life and remained throughout all of Jan's years. *De facto*, the lab was an international training center. Jan mentored over 100 graduate and postdoctoral students and visiting scientists from at least 27 different countries.


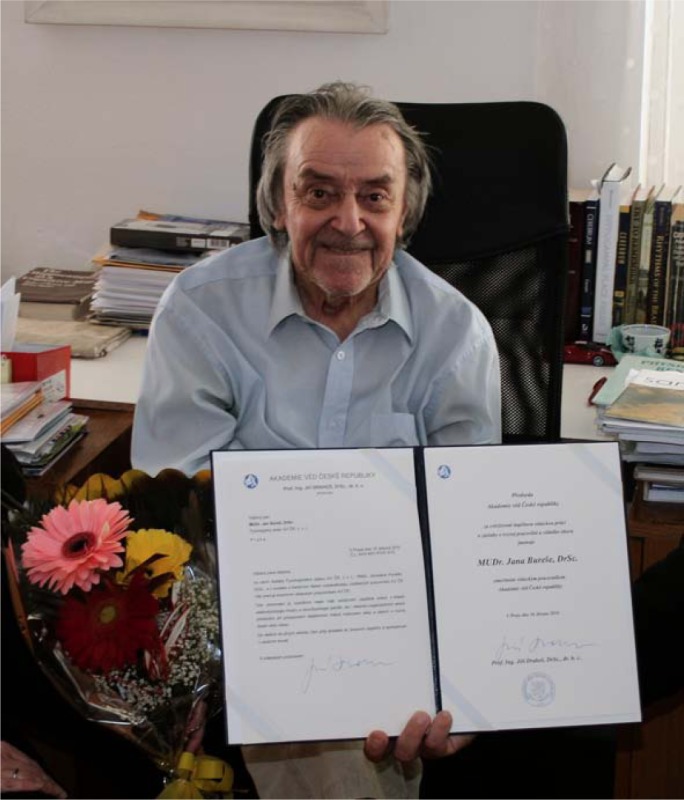


Jan Bureš was the consummate tinkerer. He invented devices, he created experimental paradigms and constructed apparatus, he developed electrophysiological techniques. He wrote important books, and published nearly 500 papers (the latest in PNAS this year). His publications ranged over topics as diverse as interhemispheric transfer of memory, conditioned taste aversion, epilepsy, and many more. Since the late 1990s Jan focused on spatial and cognitive learning in various species, including human patient populations. He has always elevated testing and eschewed theorizing. He was a passionate and masterful experimentalist.

Jan Bureš played a very important role in national and international neuroscience. He participated in the joint Soviet and USA conference that got IBRO going in the early 1960s, then served on its Central and Governing Councils until the late 1990s. He was influential in many other societies, and on the board of innumerable journals. He was a prodigious reviewer, and a tough one too.

Jan Bureš received many honors and awards in his lifetime, including election as a Foreign Associate of the National Academy of Sciences (USA). Despite his prominence, Jan was always accessible and eagerly accepted virtually every motivated student and opportunity for a new collaboration.

No summing up of Jan Bureš’ life is possible that fails to stress his humanity. He, indeed he and Olga together, lived as principled a life, as humane a life, as one can imagine in the circumstances they found themselves in. Jan Bureš was unfailingly nice, even when he was being harshly truthful. He looked for and inspired the best in people. He was a font of wisdom about science, about central Europe, about Prague, about life.

